# Difference in the level of complexity assessed on patients with advanced cancer referred to a hospital-based palliative care unit by multidisciplinary teams and wards: a retrospective study

**DOI:** 10.1186/s12913-025-12940-1

**Published:** 2025-07-01

**Authors:** Silvia Tanzi, Carlo Peruselli, Sara Alquati, Carlotta Pellegri, Simona Sacchi

**Affiliations:** 1 Palliative Care Unit, Azienda USL-IRCCS Reggio Emilia, Reggio Emilia, Italy; 2 Former President SICP, Italian Society of Palliative Care, Biella, Italy; 3 Quality Office, Azienda USL-IRCCS Reggio Emilia, Reggio Emilia, Italy

**Keywords:** Cancer, Complexity, Palliative care, Education, Appropriateness

## Abstract

**Background:**

The main criterion for the intervention of specialist palliative care is how to balance complexity of needs and prognosis. Appropriate organization and dedicated clinical tools should enable the clinicians and patients to meet this criterion.

**Methods:**

We conducted a retrospective observational study on 184 cancer patients referred to a specialized palliative care service. The difference in level of complexity was analyzed using the PALCOM scale assessment tool in regard to referrals from multidisciplinary teams versus wards.

A specialized palliative care service trained the health professionals of the multidisciplinary teams in 2019 and has been working daily in the hospital since 2013.

**Results:**

The number of referrals to the palliative care service by the wards was more than double the referrals by the multidisciplinary teams, and the level of complexity was 45% for patients referred by wards vs. 10% referred by discussion teams.

From our results, it seems reasonable to assume that training in complexity tools may increase the number of referrals to the palliative care service, while working alongside the health professionals in the wards leads to an increase in recognizing complex needs and thus better appropriateness of referrals to the palliative care service.

**Conclusions:**

A hospital-based specialist palliative care service with clinical and training expertise can increase the appropriateness of referrals.

## Background

In recent years, the field of palliative care (PC) has highlighted the importance of having increasingly reliable complexity identification tools capable of differentiating between patients who need specialist palliative care and those who can be followed by basic palliative care [1]. 

The various theories of complexity have emphasized how complexity depends not only on the “patient system” but also on the caregiver, the family in which the patient lives, the setting in which he or she is cared for, and the society in which he or she lives.

Moreover, it is not only the different characteristics of the micro-meso or macro system that affect complexity, but also the point of view of the person who is assessing that complexity, so the practitioner’s skills, the time available, and the possibility of working on a team can change the perception of complexity [2]. 

To paraphrase a famous saying: “Complexity is in the eye of the beholder.” [3].

Early introduction of palliative care and continuation throughout the course of the disease has proved to be effective in improving quality of life, appropriateness of care, and overall survival [4]. 

The increasing number of patients needing palliative care and the extended course of disease requiring palliative care impose the need for implementing palliative care in all care settings [5]. 

The main criterion for the intervention of specialist palliative care is how to balance complexity of needs and prognosis. Appropriate organization and dedicated clinical tools should enable clinicians and patients to meet this criterion.

While the perception of complexity also depends on the skills of those who see the patient with palliative care needs, it has been argued that training can improve these skills, teaching practitioners to use tools specifically designed to assess complexity [2]. In a recent paper, we described our experience of training professionals in the diagnostic therapeutic care pathways involved in the care of patients with lung, ovarian, pancreatic and lung cancer [6]. 

The training was provided by a specialized palliative care service (SPCS) within the hospital that had been in operation for about ten years. The hospital-based palliative care unit comprises a multidisciplinary team with specialized expertise in palliative care and provides consultations for in- and outpatients within the hospital. These teams are widely recognized internationally as the best model to address palliative care needs in acute settings [7]. Several studies have reported their positive impact on difficult symptom control and on reducing hospitalization and health care costs [8, 9].

The training was carried out following an analysis of the needs of the health professionals and agreements were made with them in terms of timing and modalities. Attendance was high and the numerical response in terms of consultation requests to the hospital PC unit increased.

Though the aim of the training was to increase the appropriateness of the referrals, measured by a high average level of complexity as assessed via PALCOM [10], it proved not to be sufficient to improve referral appropriateness.

The participants at the end of the training suggested to us, as an improvement intervention, to give them annual feedback on the cases sent to SPCS, whether they were appropriate.

Over the next three years all training events were discontinued due to COVID and it was only in 2022 that we were able to give feedback on the appropriateness of the referrals; we realized once again that the patients sent were not complex.

For this reason, we thought it would be interesting to compare the patients sent by the Multi-Disciplinary Teams (MDT) with the patients sent by the Wards, with whom we work on inpatient clinical cases daily.

In the present study, we compared the difference in complexity assessed via PALCOM between these two groups of patients referred to the hospital PC unit: one by the multidisciplinary teams (MDTs) we trained in 2019 and the other one from four hospital wards.

Our hypothesis was that only the training associated with continuous interaction and discussion among practitioners can lead to a change of skills and consequently of perception with respect to the complexity of palliative care needs.

To the best of our knowledge, this is the first paper to evaluate the referral to a SPCS regarding complexity screened via a specific tool in a hospital setting.

We believe that it is strategic to work on complexity (quality of the referral) with our colleagues rather than on the number of consults (quantity) to address the large amount of PC needs in hospitalized oncologic patients.

## Methods

### Context and sampling

A specialized palliative care service (SPCS) was established in Reggio Emilia (a city in northern Italy) ten years ago with expertise in clinical practice, training for health professionals (HPs), and research in palliative care.

Three physicians and two nurses are employed full-time in the unit, and it also works in close collaboration with the Psycho-oncology and Bioethics units.

The SPCS unit is located within a Comprehensive Cancer Center and a Research Institute focused specifically on oncology patients, new technologies, and new models of assistance.

The SPCS assists outpatients and inpatients, operating as a consulting team for all units in the 900-bed hospital.

To explain our volume of activity, e.g. in 2022, the SPCS performed 584 initial consultations, 299 family conferences, and 2909 total consultations (initial, follow-up and family conferences).

For the aim of this study, we retrospectively analyzed the difference in complexity between two groups of patients referred to the hospital PC unit: one by Health Professionals participating in pancreatic, lung, ovarian and hepatocarcinoma MDTs and the other one by HPs working in the Oncology, Medicine, Pneumology, Oncological Ginecology and Infectious Disease units.

Multidisciplinary discussion groups meet weekly to discuss the cases of cancer patients; they are usually focused more on surgery, radiotheraphy and chemotherapy decisions than on multidimensional patients needs ‘evaluation.

Three hundred ninety PC consultations were made in these departments in 2022.

We chose to focus on these 4 types of patients because in 2019 we did a training course in the respective Pathology Care Pathway/Multidisciplinary groups [6]; the training program consisted in theoretical lessons on assessing the physical, psychological, social, and spiritual symptoms, PROMS used in clinical practice were taught (IPOS e.g.), breaking bad news to patients and families, sharing decision making with patients and families. Theoretical lessons were completed with specialized consultations at the bed side; PC specialists performed several consultations in the department where trainees work daily in the months after the training. The Training program from a qualitative point of view, showed an increased capacity to see complexity. However, from a quantitative point of view, the patients referred to the SPCS after the intervention were not more complex patients after comparing the PALCOM evaluations of pre- and post-intervention referrals.

We retrospectively analyzed 183 charts of patients with advanced lung, ovarian, pancreatic, or hepatocellular carcinomas referred to SPCS, 123 from the wards and 61 from the MDTs.

The professionals who were involved in the training only partially overlapped with the HPs in the wards (17%, 22 physicians out of 126).

Referrals to Specialized Palliative Care Service were made by alert activation in discussion sheet during the multidisciplinary meeting; this activation was introduced after the training course in 2019.

The SPCS referral is otherwise made by the Wards via an electronic consultation request system.

Two researchers (S.T, S.S.) independently reviewed patient charts, assessing the level of complexity according to the PALCOM scale [10]. 

### Study design

This paper described a retrospective charts revision on referred patients during 2022.

We have been prospectively collecting data in a database on patients sent to us by the various services since 2015, patients divided by pathology, stage of illness, demographic data, type of service provided (physical or psychological visit, nursing follow up or phone call, family conference etc.).

For this study, the folders of 183 patients referred to the Unit were read.

To find out which patients had been referred to us by the multidisciplinary teams, a query was made to our computer system regarding the alerts triggered during the discussions.

### Data collection and analysis

Patients addressed to Palliative Care Service in 2022 were analyzed using PALCOM as the framework [10]. Italian validation of the whole instrument was not available, but singular components were already in use in clinical practice. It was composed of 5 well-known domains, measured with instruments that have been individually validated in Italian. These instruments were the performance status by Karnofsky, the physical and psychological symptoms assessment by ESAS, the Edmonton staging system for cancer pain, a checklist of general socio-familiar risk fac- tors and a list of specific ethics topics.

The difference in the PALCOM results between the 2 groups (Wards vs. MDT) were collected, as the number of PC consultations.

## Results

Sixty patients were referred to SPCS by MDTs, only 6 of whom were subsequently admitted by the service because of their complex needs.

One hundred twenty-three patients were referred by the wards, 45% of which were then treated by the SPCS.

The main reasons for assessment of medium-high complexity were the high symptom burden, socio-familial risk factors, and poor performance status.

**Fig. 1:**
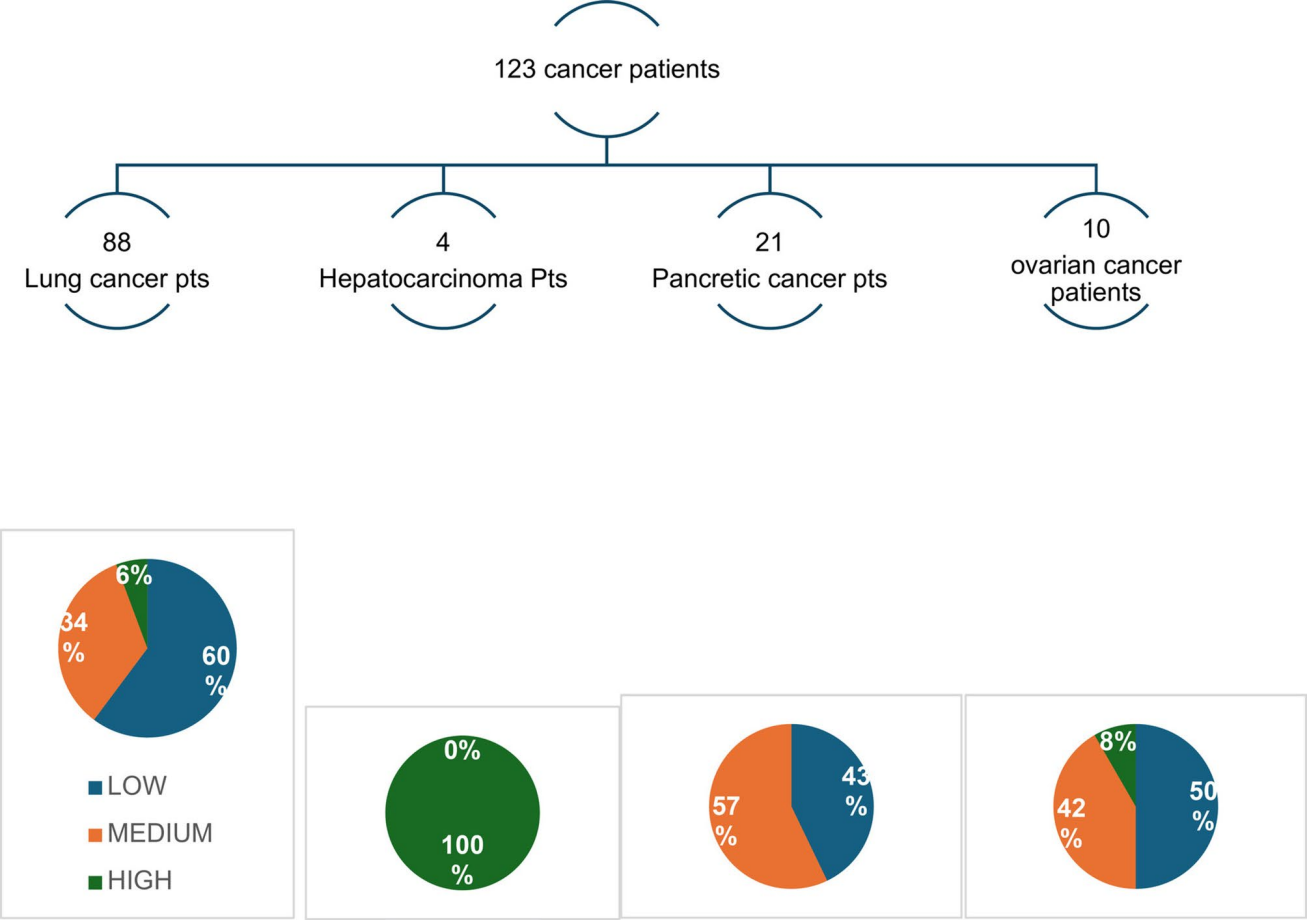
Patients referred from the Wards. Low, medium or high complexity

**Fig. 2:**
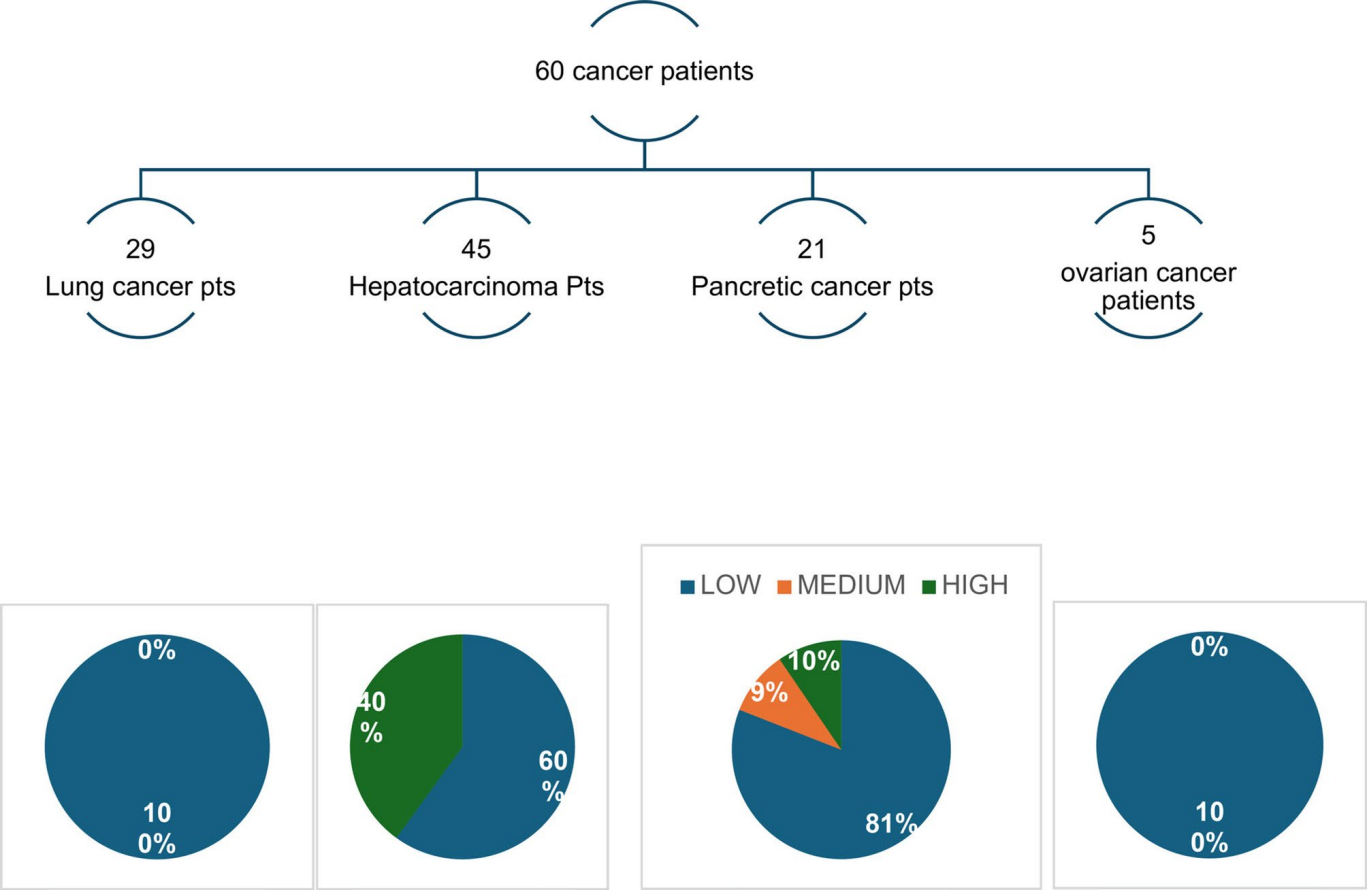
Patients referred from the Multidisciplinary Teams. Low medium or high complexity

**Fig. 3:**
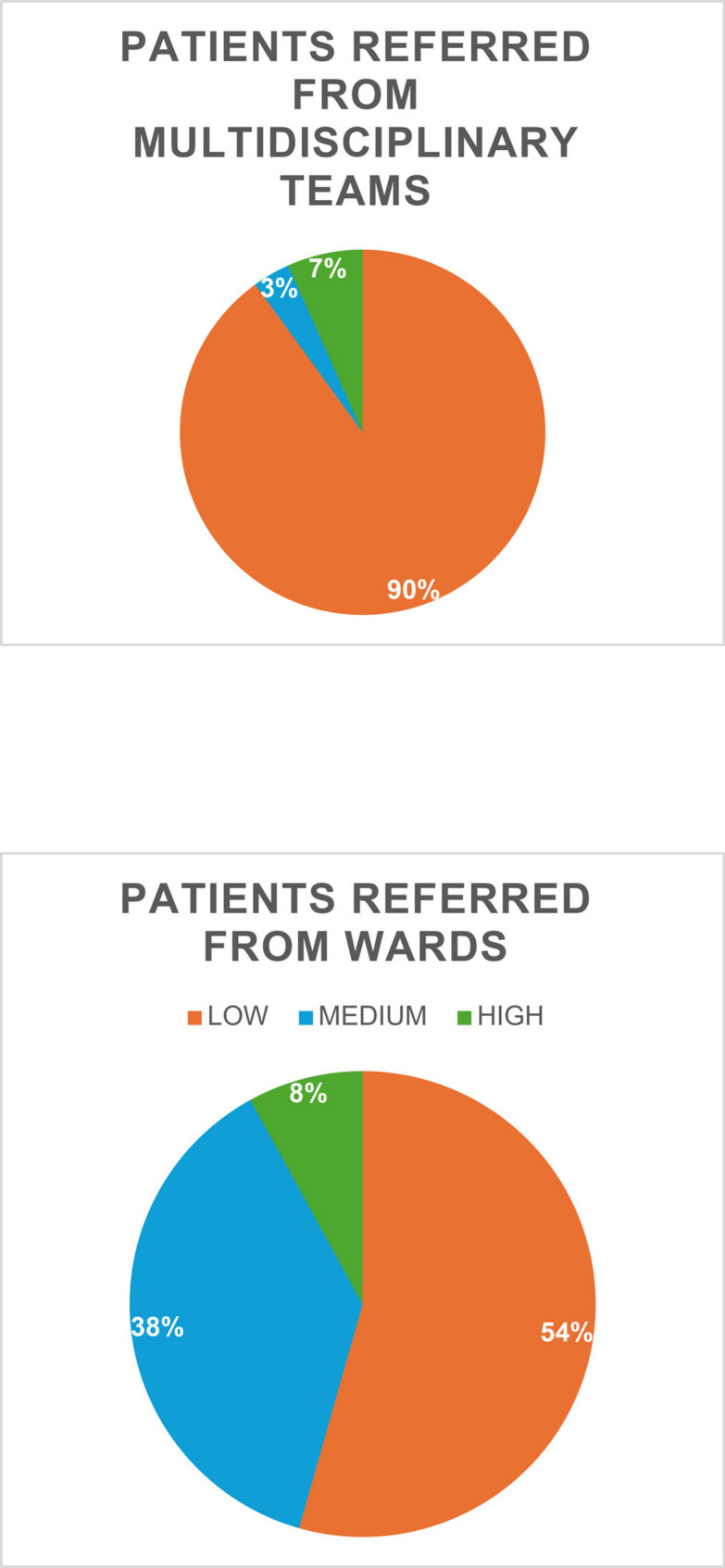
Comparison between patients referred from MDTs and patients referred from Wards. Low, medium or high complexity

**Fig. 4:**
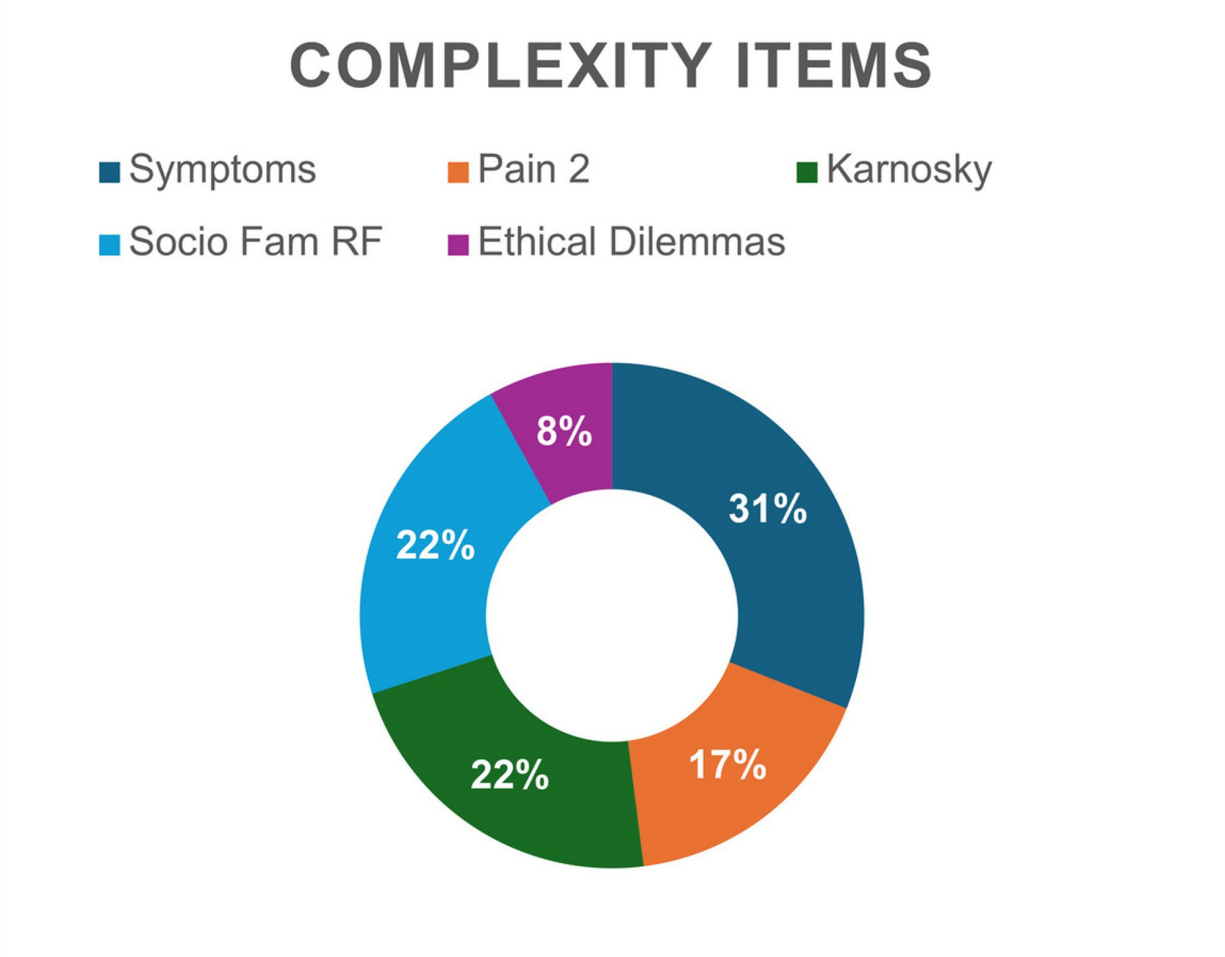
Items of complexity of patients referred from wards. Symptoms = Patients with _> 5 symptoms with NRS *≥* 4. Pain 2 = type 2 (D difficult). Karnosky ≤ 50%. Social Fam RF=-social-familial risk factors. Ethical Dilemmas = Existential and/or ethical dilemmas

Figures 1, 2, 3 and 4 better visualized and explained in details these results.

## Discussion

The number of referrals to the SPCS from the wards was more than double the referrals from the MDTs for the four cancer types. Most importantly, the level of complexity (and consequently appropriateness) was greater for patients referred by HPs working in the wards; 45% vs. 10% medium or high complexity patients referred by wards vs. discussion groups, respectively.

These results confirm our previous study on the lack of impact of a training course in PC in changing professionals attitudes, and, at the same time, confirm the impact of an SPCS operating inside a hospital, widely recognized [7, 8].

As far as we know, this is the first study on the impact on complexity achieved by a SPCS.

In our previous study [6] the participants were very active and participatory throughout the training programme and research: they all attended the course from the beginning to the end; they participated at the evaluation at the pre- and post- qualitative evaluations. The total amount of referrals increased in two out of three of the wards involved in the training, and the qualitative results suggest a deeper understanding of the palliative care role and advantages. On the contrary, we were not able see any significant increase in the complexity of the patients referred to the PC service, when comparing the measurements of the PALCOM instrument.

From our results, it therefore seems reasonable to assume that training in complexity assessment tools may increase the number of referrals to the PC service, while working alongside the HPs in the wards leads to an increase in recognizing complex needs and thus better appropriateness of referrals to the PC service.

It is also interesting to note that the Professionals who sent patients to us from the wards in only 17% of the cases were also those who had done the Training course in 2019; this could confirm the importance of daily clinical activity over the effectiveness of theoretical lessons on Palliative Care. Even Untrained Colleagues - after 10 years of Palliative Care Service within Hospital-send more numerous and more complex patients.

Could the eye of the beholder be influenced by a training program delivered by an SPCS? Or is training in the use of a screening tool alone not sufficient to help discriminate the complexity of palliative care needs and thus select the appropriateness of referrals to an SPCS?

Our data seem to confirmed that, alongside training, carefully implemented programs of colleague interaction and discussion and constant support to HPs in the wards have the potential to improve the appropriateness of referrals and more generally to improve palliative care in the hospital, as also reported by experiences published in the literature [9]. 

In this retrospective study, the items that most increase complexity are “Patients with *≥* 5 symptoms with NRS *≥* 4, low Karnosky score, and “Socio-familial risk factors”. We can suppose that the greatest impact of a hospital-based SPCS is on the presence of recognized physical symptoms and socio-familial risk.

More research is needed to improve our understanding of the effects of this combination of interventions, which components work best, and how to sustain changes in clinical practice.

In our experience, the training proposals offered on specific needs of *Entire wards* [11–14] by our in-hospital service is guaranteed to be effective; it brings changes in communication competences, ethical skills, symptom management, palliative care approach etc.

This is the reason why we are conducting trainings on Complexity ward by ward, doctors and nurses together, as an improvement project within our hospital to improve the appropriateness of referrals.

The demographic, epidemiological and social situation in Italy and Europe is constantly changing. These changes are characterized by a progressive and significant increase in the number of patients suffering from chronic degenerative diseases (cancer, cardiovascular, pulmonary, neurological pathologies, etc.) with palliative care needs, often not necessarily with a short life expectancy, due to the improvement of specific treatments for some of these diseases and for numerous forms of neoplasia.

All the international documents and studies state that faced with the increase in the number of patients with palliative care needs, it is completely unrealistic to believe that the organizational response to these needs can be based solely or even predominantly on specialist PC services. In fact, the increase in demand for palliative care already exceeds the capacity of these services to intervene, and the gap between needs and specialist resources is inevitably destined to increase in the future. For some time now, the direction supported by all the international organizations, including the WHO, and fully shared by the main palliative care scientific societies, has been to develop more “sustainable” organizational models, reserving specialist palliative care interventions exclusively for the most complex clinical and care situations.

In recent years, the organization of specialist palliative care services at the international level has been undergoing a profound change. What some authors have described as a paradigm shift has in fact led to identifying the patients who can most benefit from specialist palliative care no longer according to their prognosis but according to the complexity of their needs, regardless of diagnosis [15]. 

The validation of culturally specific tools for referring complex patients to specialist palliative care is underway all over the world, including Italy. But there is a lack of application of these tools in real contexts along with an analysis of the results. In our opinion, looking back on this retrospective analysis, it is illusory to train only on tools, even specific tools for assessing complexity. Rather, the work for appropriate referrals must be done with daily interaction and clinical discussion among the health professionals.

## Conclusions

Training alongside health professionals to identify patients with palliative care needs is mandatory in order to refer truly complex patients to specialized PC services.

A hospital-based SPCS with expertise in clinical practice and training can increase the appropriateness of referrals.

Certainly, continuous feedback to MDTs with respect to referred patients can also improve appropriateness and the efficacy of the SPCS.

Training for health professionals as well as raising awareness of society will lead to a more targeted and equitable allocation of specialist healthcare resources and consequently more personalized care for patients with palliative care needs.

## Data Availability

The study documentation is collected and managed by the coordinator of the study centre (PC Unit, AUSL – IRCCS di Reggio Emilia), and datasets are available on reasonable request.

## Abbreviations

MDT: Multidisciplinary team
SPCS: Specialized Palliative Care Service
PC: Palliative Care
Hp: Health Professionals
KPS: Karnosky Performace Score
WHO: World Health Organization
